# Penetrating cardiac injury

**DOI:** 10.1186/1749-8090-8-S1-P41

**Published:** 2013-09-11

**Authors:** PL Tahalele, Puruhito, A Prasmono, H Kusbijanto, H Soebroto, YE Sembiring

**Affiliations:** 1 Department of Surgery Division of Cardiothoracic and Vascular Surgery School of Medicine Airlangga University - Dr. Soetomo General Hospital Surabaya, Indonesia

## Background

Penetrating cardiac injury has the highest mortality and morbidity rates of all organ injury. The prehospital mortality rate for penetrating cardiac injury was 70 – 80%. Prehospital care including rapid transportation of patients to trauma center and agresive intervention has increased survival rate.

The purpose of this study was to show our experience in treatment of patient with penetrating injury to the cardiac.

## Methods

This is retrospective studies from 1985 to 2011 (26 years) at emergency room Dr. Soetomo General Hospital reporting of 47 patients with penetrating injury to the cardiac.

## Results

In this review we have defined 47 patients with male 36 patients (76.59 %) and female 11 patients (23.40 %). Age is ranged from 9-64 years. The mechanism of injury consist: stab wound (knife, stiletto) 35 patients (74.47 %), air rifle (bullet) 5 patients (10.64 %), segment fracture of the rib one patient (2.12 %) and CVP catheter one patient (2.12). The injured site are Right Ventricle 30 patients (63.83 %), Left Ventricle 5 patients (10.63 %), Right Atrium 4 patients (8.51 %), Ascending Aorta one patients (2.12 %), and Pulmonary Artery 1 patients (2.12 %).

There were 5 major complications: icterus in 14 patients (29.78 %), wound dehiscent in one patient (2.12 %), sepsis in one patient (2.12 %), VSD in one patient (2.12 %), mortality in 5 patients (10.63 %). The surviving is 42 patients (89.36 %).

## Conclusion

The result of our experience has been satisfactory in all cases. Because of this experience, we recommend aggressive thoracotomy exploration to lower operative mortality and morbidity.

